# Living the Good Life? Mortality and Hospital Utilization Patterns in the Old Order Amish

**DOI:** 10.1371/journal.pone.0051560

**Published:** 2012-12-19

**Authors:** Braxton D. Mitchell, Woei-Jyh Lee, Magdalena I. Tolea, Kelsey Shields, Zahra Ashktorab, Laurence S. Magder, Kathleen A. Ryan, Toni I. Pollin, Patrick F. McArdle, Alan R. Shuldiner, Alejandro A. Schäffer

**Affiliations:** 1 Department of Medicine, Program for Personalized and Genomic Medicine, University of Maryland School of Medicine, Baltimore, Maryland, United States of America; 2 National Center for Biotechnology Information, National Library of Medicine, National Institutes of Health, Department of Health and Human Services, Bethesda, Maryland, United States of America; 3 Department of Epidemiology & Public Health, Program for Personalized and Genomic Medicine, University of Maryland School of Medicine, Baltimore, Maryland, United States of America; 4 Geriatric Research and Education Clinical Center, Veterans Administration Medical Center, Baltimore, Maryland, United States of America; Foundation for Liver Research, United Kingdom

## Abstract

Lifespan increases observed in the United States and elsewhere throughout the developed world, have been attributed in part to improvements in medical care access and technology and to healthier lifestyles. To differentiate the relative contributions of these two factors, we have compared lifespan in the Old Order Amish (OOA), a population with historically low use of medical care, with that of Caucasian participants from the Framingham Heart Study (FHS), focusing on individuals who have reached at least age 30 years.

Analyses were based on 2,108 OOA individuals from the Lancaster County, PA community born between 1890 and 1921 and 5,079 FHS participants born approximately the same time. Vital status was ascertained on 96.9% of the OOA cohort through 2011 and through systematic follow-up of the FHS cohort. The lifespan part of the study included an enlargement of the Anabaptist Genealogy Database to 539,822 individuals, which will be of use in other studies of the Amish. Mortality comparisons revealed that OOA men experienced better longevity (p<0.001) and OOA women comparable longevity than their FHS counterparts.

We further documented all OOA hospital discharges in Lancaster County, PA during 2002–2004 and compared OOA discharge rates to Caucasian national rates obtained from the National Hospital Discharge Survey for the same time period. Both OOA men and women experienced markedly lower rates of hospital discharges than their non-Amish counterparts, despite the increased lifespan.

We speculate that lifestyle factors may predispose the OOA to greater longevity and perhaps to lesser hospital use. Identifying these factors, which might include behaviors such as lesser tobacco use, greater physical activity, and/or enhanced community assimilation, and assessing their transferability to non-Amish communities may produce significant gains to the public health.

## Introduction

Average lifespan in the United States (US), as in many other developed countries throughout the world, has been increasing over time [1]. Within the US, more economically advantaged persons live longer [2]. This advantage that has been attributed to multiple factors, including: better access to high quality medical care and a lower rate of tobacco use. In particular, the death rate from cardiovascular events has decreased dramatically over the past 30 years [3]. The decrease in cardiovascular deaths has been attributed in part to improvements in rushing heart attack sufferers to the hospital and administering life-saving emergency treatments both in the ambulance and in the emergency room [4], [5]. Changes such as cholesterol-lowering drugs, decreases in smoking and heart healthier diets, are also important contributors.

The relative contributions to lifespan made by improvements in medical care access and technology versus improvements in lifestyle have been hard to quantify. This is an important issue in public policy given that lifespan in the US lags behind that of many other developed nations despite health care costs in the US, estimated at $2.6 trillion in 2010 [6], that dwarf those in other countries.

To address this issue, we have estimated lifespan in the Old Order Amish (OOA), a subpopulation within the US with low utilization of specialized health care. The OOA do not participate in government-sponsored plans, such as Medicare and Medicaid, and are generally self-insuring as a group. Access may be further limited because OOA do not own cars and may find it difficult to reach locations where specialized health care is delivered. Although they avoid most forms of modern technology, the OOA do seek modern health care and hospitals, as we quantify in this study.

Concurrent with their reduced access to health care and especially high technology health care, the OOA lifestyle is distinctive in other ways, some of which may be health-promoting. Especially notable is the degree to which Amish core beliefs in church, community, and selflessness permeate daily life. Compared to non-Amish, Amish are very physically active; they maintain a traditional lifestyle, still utilizing the horse and buggy as their main mode of transportation and do not use electricity in their homes. The Amish also differ from the non-Amish in several other lifestyle factors, including smoking habits and cardiovascular disease risk.

Gaining a more complete picture of the medical utilization patterns and overall health of the OOA may be revealing. In this report, we characterize mortality rates and hospital discharge rates in the Amish and contrast them with those in non-Amish Caucasians.

## Methods

This report is based on the OOA community living in Lancaster County, PA, whom we have been studying since 1993. This community was founded by hundreds of individuals who immigrated to this area from central Europe during the early 18^th^ century, with the present day Lancaster County OOA community comprised of their descendants. Our analysis plan consisted of two parts. First, we used a newly enlarged Anabaptist Genealogy Database Version 5 (AGDB5) to identify a birth cohort of 2,108 OOA born between 1890–1921 who lived until age 30 years or older and compared mortality in these individuals to that in Caucasian participants of the Framingham Heart Study (FHS). Second, we compared hospital discharge rates between OOA and US Caucasians from the National Hospitalization Discharge Survey (NHDS). To find suitable denominators, we used published registry data to compile a census of all OOA residing in Lancaster County in 2002. To find the corresponding numerators, we identified all OOA discharges from Lancaster county area hospitals from 2002–2004 to provide estimates of hospital discharge rates.

### Mortality in OOA vs FHS

#### Amish mortality cohort

We identified a cohort of OOA individuals born between 1890 and 1921 whose vital status we ascertained. This Amish mortality cohort comprised ancestors and their siblings of the 4,200 OOA individuals who had participated in one or more of the population studies carried out in this community from 1995–2010 by our group [7]–[10]. By using subjects we had studied, we assured that they were practicing OOA living in Lancaster County. Identification of the relatives was made possible by accessing the AGDB, a computer-searchable genealogy database of the OOA and other Anabaptist populations dating back to the OOA immigration to the US that was generated and is managed by co-author AAS and colleagues at the NIH. There are multiple versions of AGDB [11]–[15] constructed using various sources and updates. The mortality part of the study included the expansion of AGDB to include major updates of two of the sources and to increase the size to 539,822 individuals (see Information S1). Figure S1 shows the overlaps of the three sources used to construct AGDB5.

The characterization of a cohort from AGDB5 to study mortality is illustrated schematically in Figure S2. From AGDB5, we identified a total of 9,778 ancestors and their siblings, of whom 2,259 survived until at least age 30 years and were born between 1890 and 1921. We excluded subjects with missing death dates who had a recorded birth place outside of Lancaster County (n = 151). Of the 2,108 remaining individuals, 121 were born in Lancaster County but had no date of death. Further review and fieldwork by our staff of research nurses and Amish liaisons identified dates of death for 11 of these 121 subjects and confirmed 45 others to be alive as of January 1, 2011. The remaining 65 subjects (13 reported as deceased but with unknown year of death, and 52 with unknown vital status) were excluded from our analysis. Mortality follow-up was thus complete on 96.9% (2,043/2,108) of the cohort (see Information S1 for additional detail).

#### FHS mortality cohort

The FHS is a longitudinal study of factors influencing the development of cardiovascular disease [16]. The original FHS cohort comprised 5,079 subjects, born between 1887 and 1921, who were aged 28–62 years at enrollment into the study in 1948–1952. All were invited back every two years for a repeat examination. We obtained the 2012 release of the FHS-Cohort dataset with vital status follow-up through 2007 from the NHLBI BioLINCC website (https://biolincc.nhlbi.nih.gov/). Through 2007, 4,745 of the original FHS participants had died (93.4%) and had available death dates. Participants with no death date recorded (n = 334) were censored as of date of last contact.

#### Mortality comparison

The Amish entered the mortality cohort on the day of their 30th birthday. FHS participants entered on the day of their baseline FHS visit. We used proportional hazards regression analysis to compare the survival functions between the two cohorts. Analyses were carried out in men and women separately using the PHREG procedure of the SAS software package (Cary, NC).

### Amish Census

We estimated the population size of the Lancaster County Amish community using residence information obtained from the 2002 Church Directory of the Lancaster County Amish [17]. In 2002, the Amish Lancaster community included 141 distinct OOA church districts, of which 132 are situated in Lancaster County. Each district comprises 17–50 households (mean households/district = 30) [18]. The Church Directory enumerates for each district all household members and their birthdates. We sampled 30 districts (23%), selected at random, enumerated the number of OOA individuals by age and sex, and then extrapolated the numbers of OOA in all 132 districts in Lancaster County. More details are in the Information S1 and in Table S1.

### Hospital discharge survey

We carried out a population-based ascertainment to identify all hospital discharges (deceased or alive) among the OOA from Lancaster County for the 3-year period January 1, 2002 to December 31, 2004. We previously used this approach to estimate rates of hip fractures in the Amish [19]. Discharges were recorded via a review of the medical records from the four area hospitals in the Lancaster County area that encompass the likely encatchment area for the OOA (see Figure S1).

We restricted our analysis of discharge records for OOA individuals age≥25 years, to focus on adults. Patients were identified by the hospitals as Amish either because they had specified their religious affiliation as Amish or had indicated participation in the Amish Aid plan as their means of health insurance. We verified OOA religious affiliation and residency in Lancaster County by cross-referencing patient names with the OOA Church Directory, which contains only OOA (vs other Amish groups), and/or verification with an Amish research liaison. Out of 658 OOA discharges identified by the four hospitals, 181 did not meet validation (i.e., lived outside of Lancaster County or were not OOA), leaving 477 confirmed OOA discharges. For each discharge, we recorded the principal diagnosis and up to 6 additional discharge diagnoses. Discharges were coded using ICD-9-CM codes.

Hospital discharge rates in the OOA were determined by dividing the number of discharges by the population at risk and were compared with those in Caucasians from the NHDS for years 2002–2004. The NHDS, conducted since 1965, represents the main source of national data on characteristics of patients discharged from US non-federal short-stay hospitals. The survey collects data from a sample of medical records obtained from a nationally representative sample of short-stay, general hospitals. We compared rates between OOA and NHDS using the indirect method for age-adjustment [20]. Specifically, we used the NHDS discharge rates to compute the expected number of discharges in the OOA had this group experienced the same rates as those observed from the NHDS.

### Ethics Statement

The study was approved by the University of Maryland Institutional Review Board. Informed consent was not obtained; the mortality component of the study was based on publicly available data maintained by NIH through the AGDB (Amish) and NHLBI BioLINCC Biorespository (Framingham Heart Study), and a waiver for informed consent was granted by the Directors of Research from Lancaster Regional Medical Center, Ephrata Community Hospital, and Ephrata Community Hospital because the study involved a review of medical records only, did not require collection of personal identifying information, and the research could not be reasonably conducted without a waiver of consent.

## Results

### Mortality Comparison

Of the 2,043 subjects in the OOA mortality cohort (birth years 1890–1921), 1,998 were known to be deceased (97.8%) by 2010 and the remaining 45 confirmed as alive through January 1, 2011. Ages at death of the 1,998 deceased subjects ranged from 30–103 years. By virtue of their participation in FHS (birth years 1887–1921), all subjects lived until at least age 30 years. Ages at death among the 93.4% of the FHS cohort in whom death dates were reported ranged from 32–104 years.

**Figure 1:**
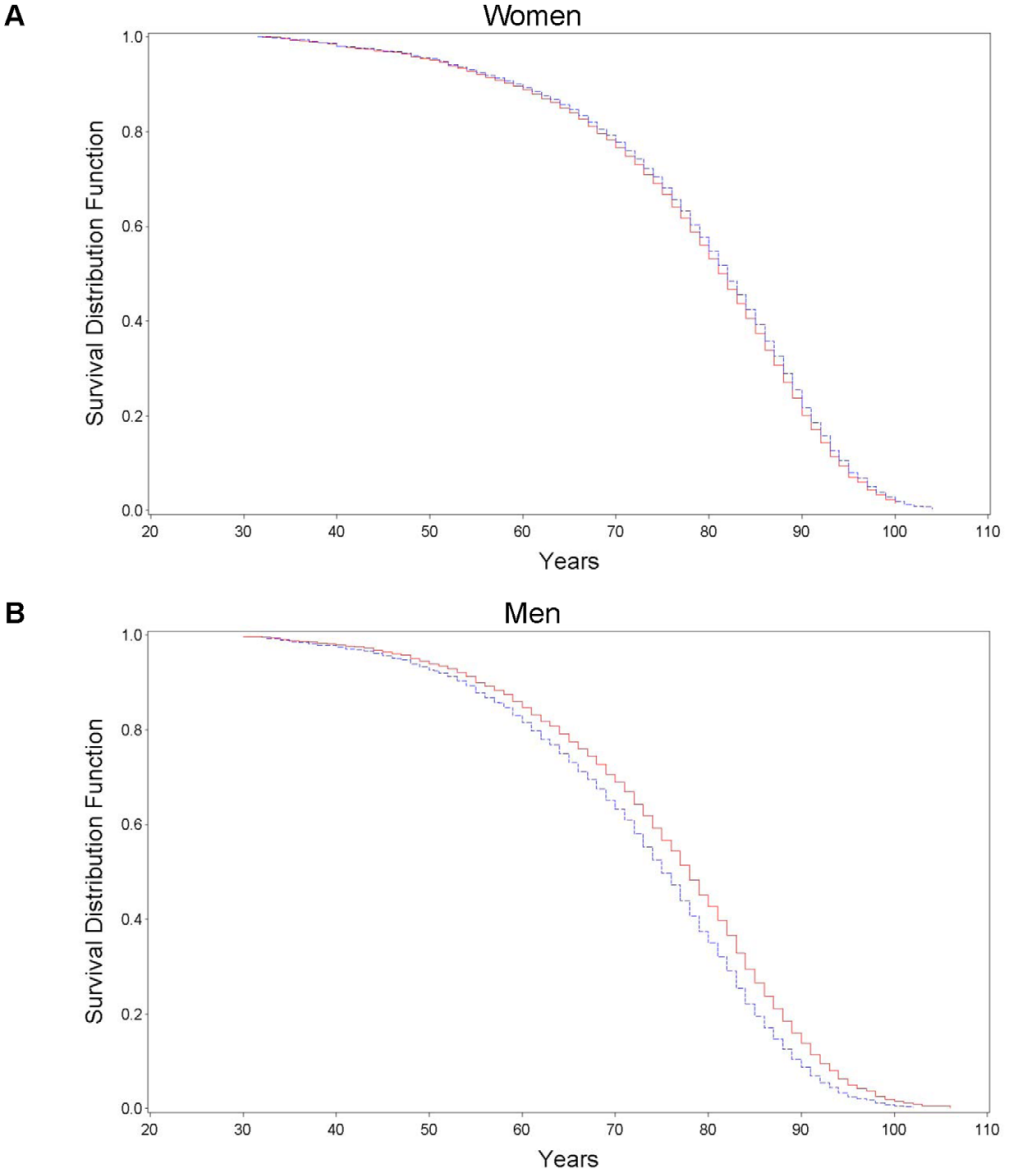
Survival curves of Old Order Amish (red solid line) and Framingham Heart Study (blue hatched line) among those surviving until at least age 30 years.

Figures 1a and 1b show the survival curves for OOA and FHS women and men, respectively. Amish men were expected to live on average 3 years longer than their FHS counterparts (hazard ratio 1.23, 95% confidence intervals 1.14–1.32; p<0.001), while there was virtually no difference in expected life expectancy between OOA and FHS women (hazard ratio 0.95, 95% confidence intervals 0.88–1.02; p = 0.15).

### Hospital discharge rates in the OOA and comparison with US Caucasians

From the 2002 Church Directory of the Lancaster County Amish, we estimated there to be ∼21,850 OOA individuals residing in Lancaster County, PA, including ∼7,502 aged 25 years and older. (see Table S1 for estimated age and sex distribution of OOA residents of Lancaster County.)

**Figure 2:**
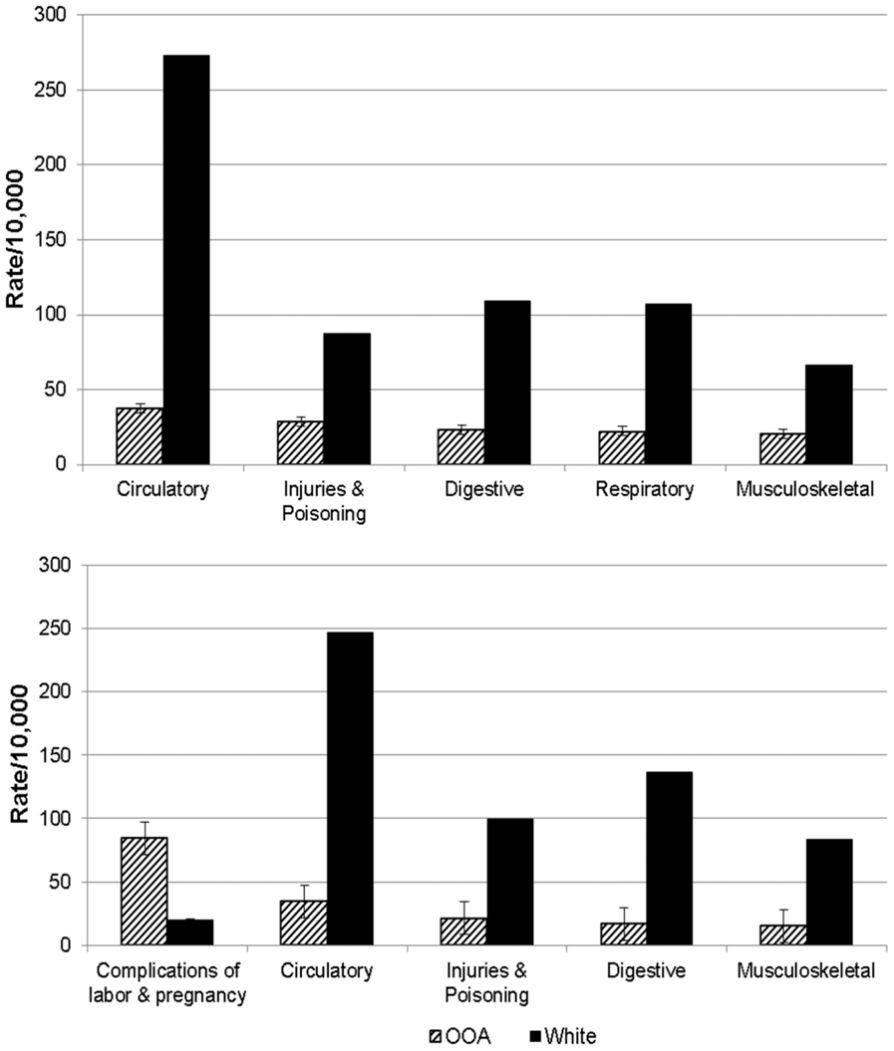
Annualized rates of first-listed hospital discharges in Lancaster County Old Order Amish (OOA) (solid bar) and US Caucasians (striped bar) for ages 25 years and older. The five leading diagnostic categories in the OOA are shown. Non-Amish white hospital discharge rates from NHDS. OOA and US Caucasian hospital discharge rates obtained for the period 2002–2004 and annualized. Men are in the upper panel and women are in the lower panel.

Annualized rates of first- and any-listed hospital discharges for each diagnostic category are shown in Table 1 for the OOA and for the US Caucasian population. OOA and US Caucasian men shared four of the top five diagnostic categories for first-listed hospital discharges (circulatory, injuries/poisoning, digestive, and respiratory), and OOA and US Caucasian women shared three of the top five diagnostic categories (circulatory, injuries/poisoning, and digestive). In the five leading diagnostic categories observed in OOA, first-listed discharge rates were substantially lower in OOA than in US Caucasians, with the exception of complications of labor, childbirth, and the puerperium, which were higher in OOA compared to US Caucasian women (Figures 2a and 2b). See Tables S2, S3, S4, S5 for age-specific comparisons of first- and any-listed hospital discharge rates between OOA and US Caucasians.

**Table 1:** Annualized rates of first-listed and any-listed hospital discharges in the Lancaster County Old Order Amish and in US Caucasians from the National Health Discharge Survey (2002–2004), ages 25 years and older (expressed as # discharges per yr/10,000 people).

	First listed discharges				Any listed discharges			
	Men		Women		Men		Women	
Diagnosis (ICD-9-CM codes)	Old Order Amish	US Caucasians	Old Order Amish	US Caucasians	Old Order Amish	US Caucasians	Old Order Amish	US Caucasians
Infectious and parasitic diseases (001–139)	0.9	24.8	0.9	30.1	25.8	117.9	12.4	142.1
Neoplasms (140–239)	16.0	52.8	9.8	73.0	38.2	155.1	28.5	191.3
Endocrine, nutritional and metabolic diseases, and immunity disorders (240–279)	4.4	44.9	1.8	64.6	95.0	602.6	88.9	786.5
Diseases of the blood and blood-forming organs (280–289)	3.6	10.1	0.0	15.0	50.6	137.7	31.1	193.8
Mental disorders (290–319)	7.1	70.5	1.8	68.2	37.3	369.2	12.4	409.6
Diseases of the nervous system and sense organs (320–389)	0.9	14.6	0.0	20.2	17.8	117.4	13.3	152.8
Diseases of the Circulatory System (390–459)	37.3	273.3	34.7	246.6	203.4	1366.8	182.3	1384.6
Diseases of the respiratory system (460–519)	22.2	107.1	10.7	128.1	75.5	396.7	32.9	446.6
Diseases of the digestive system (520–579)	23.1	109.4	16.9	136.3	56.8	343.2	42.7	450.0
Diseases of the genitourinary system (580–629)	0.4	44.3	13.3	93.2	26.6	234.7	56.9	388.2
Complications of pregnancy, childbirth, and the puerperium (630–679)	0.0	0.0	84.5	20.3	0.0	0.0	211.6	443.8
Diseases of the skin and subcutaneous tissue (680–709)	1.8	22.2	3.6	21.1	5.3	80.6	10.7	83.8
Diseases of the musculoskeletal system and connective tissue (710–739)	20.4	66.6	15.1	83.3	48.0	165.2	42.7	312.0
Congenital anomalies (740–759)	1.8	1.7	0.9	2.4	2.7	6.9	2.7	8.0
Certain conditions originating in the perinatal period (760–779)	0.0	0.1	0.0	0.2	0.0	0.6	0.0	0.5
Symptoms, signs, and ill-defined conditions (780–799)*	14.2	6.9	4.4	8.0	44.4	249.3	41.8	309.1
Injury and poisoning (800–999)	28.4	87.3	21.3	99.6	57.7	218.8	32.0	217.5
Supplementary classification (V01–V91)**	8.9	29.7	0.9	221.2	91.5	309.6	132.5	542.1

* Symptoms = alteration of consciousness, hallucinations, syncope and collapse, convulsions, dizziness, sleep disturbances, fever, malaise and fatigue, hyperhidrosis and other general symptoms;

** Supplementary = potential health hazards related to different personal and family circumstances, and health services encountered for different reasons including birth.

**Figure 3:**
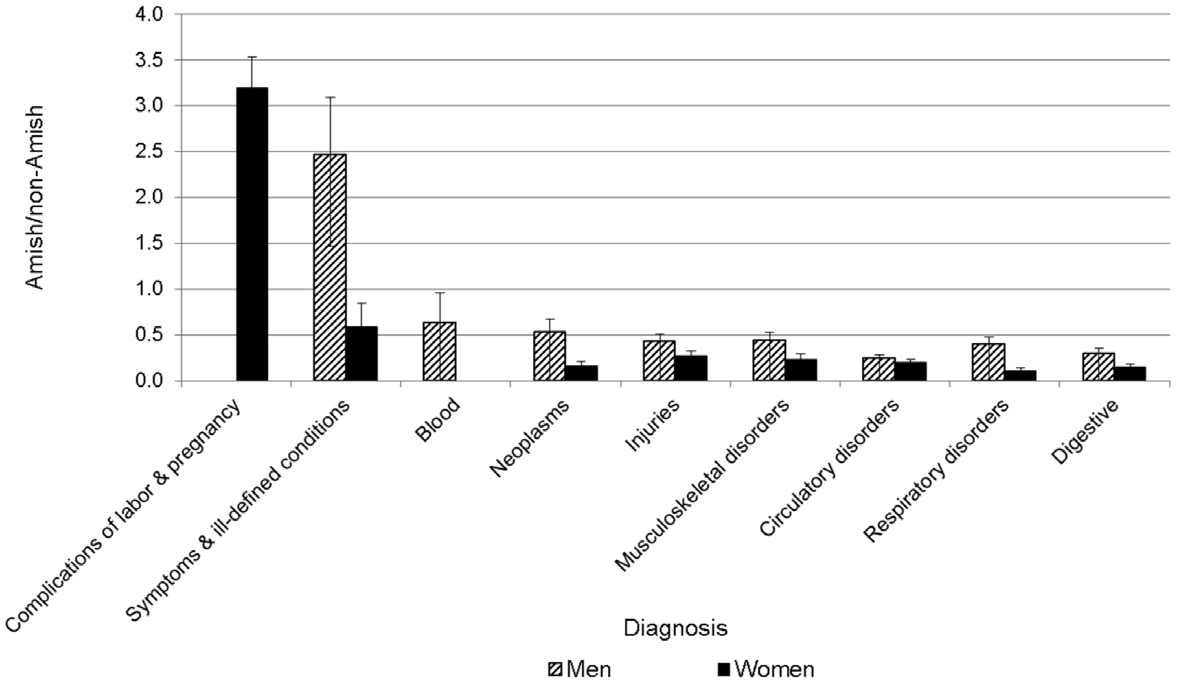
Excess (or deficit) of first-listed hospital discharges in the OOA compared to US Caucasians. OOA and US Caucasian hospital discharge rates obtained for the period 2002–2004 and annualized. The standard error of the observed (O) vs. expected (E) ratio computed as (√O)/E).

When adjusting for age differences between the two populations, OOA men and women experienced substantially lower rates of first-listed (Figure 3) and any-listed (Information S1) of hospital discharges for the major categories (p<0.05 for each comparison), with the exceptions of a 3.2-fold greater excess (95% confidence interval: 2.55–3.85) among OOA women of first-listed hospital discharges for complications of labor and pregnancy, and a 2.5-fold excess (95% confidence interval: 1.25–3.68) of first-listed hospital discharge rates among OOA men for supplemental conditions. Since OOA women have on average three times as many children as US Caucasian women (∼7 children per OOA mother on average), then the hospital discharge rates normalized for number of births are similar between OOA and US Caucasian women. The ratios comparing hospital discharge rates are in Table S6.

## Discussion

Our analyses reveal that OOA men experience better longevity than Caucasian men from the FHS and OOA women experience comparable longevity, despite a much lower rate of hospitalizations. This is a remarkable observation in light of escalating costs of medical care in the US. That OOA have reduced usage of medical care has been noted anecdotally [21], [22], but to our knowledge, has not previously been quantified systematically. Our extensive interactions with the OOA community over the past 18 years suggest that the reduced OOA usage of health care is primarily due to cultural norms regarding when to seek medical care due in part to community refusal to use health insurance, and secondarily to limits on transportation.

That OOA adults have better or similar longevity compared to non-Amish Caucasian adults was first reported over 30 years ago by Hamman et al., who observed from their analysis of death certificates that OOA men experience lower mortality rates than non-Amish residents of the same counties. In that study, there were no significant differences in mortality between OOA women and non-Amish women at ages 40–69 years, and higher mortality rates among OOA women over age 69 years [23]. The survival advantage experienced by OOA men was due principally to lower rates of cancer and cardiovascular diseases. Our study updates and extends Hamman's earlier study in two important ways, first by focusing on a more contemporaneous period of study and second by computing lifespan directly, allowing for direct comparison with the FHS. Our cohort analysis is thus protected against potential biases inherent in death certificate studies that can arise from incomplete ascertainment of death certificates or under/over-estimation of the population.

We quantified a dramatically lower hospitalization discharge rate in OOA compared to the US Caucasians. The lower rate among the OOA is evident across a wide spectrum of diagnostic categories. While we likely undercounted discharges for mental conditions by not including regional specialty behavioral clinics in our survey, we acknowledge that it is also possible that we may have undercounted the number of OOA discharges at the primary hospitals since they were identified on the basis of self-reported religious affiliation or use of the Amish Aid health plan. It is further possible that some Amish may have received medical care in hospitals outside of the Lancaster County area. A modest undercount would not have materially affected our conclusions since for most diagnostic categories the US Caucasian hospital discharge rates were 3–10 times higher than those in the OOA. While hospital discharge rates for complications of labor, childbirth, and the puerperium were approximately 4 times higher among OOA compared to US Caucasian women, they were more or less comparable after considering the 3–4-fold higher number of children observed among OOA women. Our study cannot address whether the lower hospitalization discharge rates of the OOA indicate that the OOA have less need of hospital care than non-Amish because they are healthier, or whether greater access to hospitals would improve lifespan further. Indeed, these two possibilities are not mutually exclusive.

We speculate that lifestyle factors may predispose the OOA to better health, lesser need for hospitalizations, and greater longevity. But which components? We have previously compared traditional cardiovascular risk factors between OOA and non-Amish Caucasians and shown that while OOA have less abdominal obesity (as measured by waist circumference) despite comparable body mass index compared to non-Amish (28.4 vs 28.5 kg/m^2^), they also have a less advantageous lipid profile as indicated by significantly higher total (5.5 vs 5.2 mmol/L) and LDL (3.59 vs 3.04 mmol/L) cholesterol levels [24]. Diastolic blood pressure levels are also significantly higher in OOA individuals (72.0 vs 70.4 mmHg), and the OOA are much less likely to take lipid-lowering (3.7% vs 22.9%) or blood pressure-lowering (6.2% vs 22.5%) medications compared to their non-Amish counterparts.

Despite the overall less beneficial cardiovascular risk factor profile of OOA in terms of lipids and blood pressure, OOA have more favorable patterns of several other important cardiovascular risk factors. First, OOA are on average considerably more physically active than non-Amish. In a recent study, we measured physical activity levels by accelerometry in OOA and non-Amish children and found OOA children to be 3.3 times less likely to be overweight than non-Amish children and physical activity levels to be substantially higher in the OOA children, with boys more active than girls in both groups, but OOA girls easily more active than non-Amish boys [25]. Moreover, despite comparable levels of BMI, we have also previously reported the prevalence of type 2 diabetes is ∼50% lower in the OOA compared to US Caucasians [7], a result we have speculated may be due to higher physical activity levels [26]. We have similarly documented high levels of physical activity in Amish adults [27].

Second, rates of cigarette smoking are significantly lower in the OOA than non-Amish [10], [28], with ∼20% of OOA men reporting that they smoke and virtually no women. Moreover, among OOA reporting that they smoke, the intensity of smoking tends to be low (i.e., most smokers report weekly cigar smoking). According to the Centers for Disease Control, adults who smoke cigarettes on average, die 14 years earlier than nonsmokers [29]. Approximately 52% of Framingham Heart Study subjects reported themselves as smokers during their baseline visit [30] and assuming a 10% overall smoking rate in OOA, then we crudely estimate that the reduced level of smoking in the OOA could account for as much as a 5.9 yr longer lifespan as compared to FHS participants (calculated as (0.52–0.10) ×14 years).

Third, the role of sociocultural factors in health and disease is complex, but the very cohesive nature of the OOA community, including its strong social support network may provide significant benefits to health and recovery. Almost all elderly Amish are cared for by their families with assisted care or nursing home placements very uncommon. The health and lifespan enhancing benefits of social relationships are speculated to operate indirectly by moderating or buffering the deleterious influence of stressors on health and/or promoting the development of individual cognitive, emotional, and behavioral skills [31]. In a recent meta-analysis of over 300,000 subjects, components of social relationships were associated with decreased mortality, and notably the magnitude of the effect was on par with that of quitting smoking and exceeded those associated with other large risk factors such as obesity and physical inactivity [32].

In addition to environmental and lifestyle factors, it is also possible that differences in genetic background between OOA and FHS participants contribute to differences in their mortality experience. The genetic background of the OOA is unique in the sense that the Lancaster settlement represents a founder population such that virtually all 30,000 OOA current residents are descendants of a few hundred founders who immigrated to the region in the early 1700's [33]. As a consequence of this history, the OOA gene pool is relatively defined, resulting in a relative excess in the frequency of consanguineous marriages. Renowned geneticists have tried to estimate the deleterious effects of inbreeding for over 50 years and there is evidence of increased mortality in children and young adults, but these studies of inbreeding have been unable to estimate any effects on post-reproductive mortality and longevity [34], [35]. However, there is at least some evidence that inbreeding is associated with increased cardiovascular risk in adulthood, including higher levels of blood pressure and cholesterol levels [36].

In the past 30 years in the US, the cost of health care has increased dramatically and much faster than the rate of inflation [37]. Despite these costs, OOA adults, who use hospitals at a far lower rate than non-Amish, experience comparable or better lifespan than their non-Amish counterparts. From this perspective, our data highlight the need to more fully understand the factors that contribute to fewer hospitalizations in OOA adults. The benefits of such factors at the population level could be larger than the incremental gains associated with improvements in medical care access. Our results suggest that interventions targeted at lifestyle factors may have higher impact on improving lifespan at the population level than improvements in medical technology and medical care access. Identifying these factors, and assessing their transferability to non-Amish communities may produce significant gains to the public health. The expansion of AGDB to version 5 is already proving useful in other studies of the Amish where relationship information is needed (e.g., [38]).

## Supporting Information

Figure S1 **Lancaster County, including locations of Lancaster General Community Hospital (1), Lancaster Regional Medical Center (2), Ephrata Community Hospital (3), and Ephrata Community Hospital (4).** From http://www.lancasterhistory.org/images/stories/lancaster_county_history/map_townships.jpg.
(TIF)

Figure S2 **Construction of the OOA mortality cohort.**
(TIF)

Table S1 Age and sex distribution of the Lancaster County Old Order Amish population as of July 1^st^ 2002 * (percentages in parentheses).
(DOCX)

Table S2 **Three-year rates of first-listed hospital discharges (per 10,000) among Old Order Amish residing in Lancaster County, Pennsylvania, 2002–2004.**
(DOCX)

Table S3 **Three-year rates of first-listed hospital discharges (per 10,000), Caucasians from the NHDS, 2002–2004.**
(DOCX)

Table S4 **Three-year rates of any-listed hospital discharges (per 10,000) among Old Order Amish residing in Lancaster County, Pennsylvania, 2002–2004.**
(DOCX)

Table S5 **Three-year rates of any-listed hospital discharges (per 10,000), Caucasians from the NHDS, 2002–2004.**
(DOCX)

Table S6 Standardized ratios* comparing hospital discharge rates for any-listed diagnostic procedures between Old Order Amish and non-Amish whites, 2002–2004. Non-Amish white rates from the NHDS.
(DOCX)

Information S1 (DOCX)
