# Supplementary figures and images for: Living the Good Life? Mortality and Hospital Utilization Patterns in the Old Order Amish
Source: PLoS One. 2012 Dec 19;7(12):e51560. doi: 10.1371/journal.pone.0051560 (PMC3526600; doi:10.1371/journal.pone.0051560)

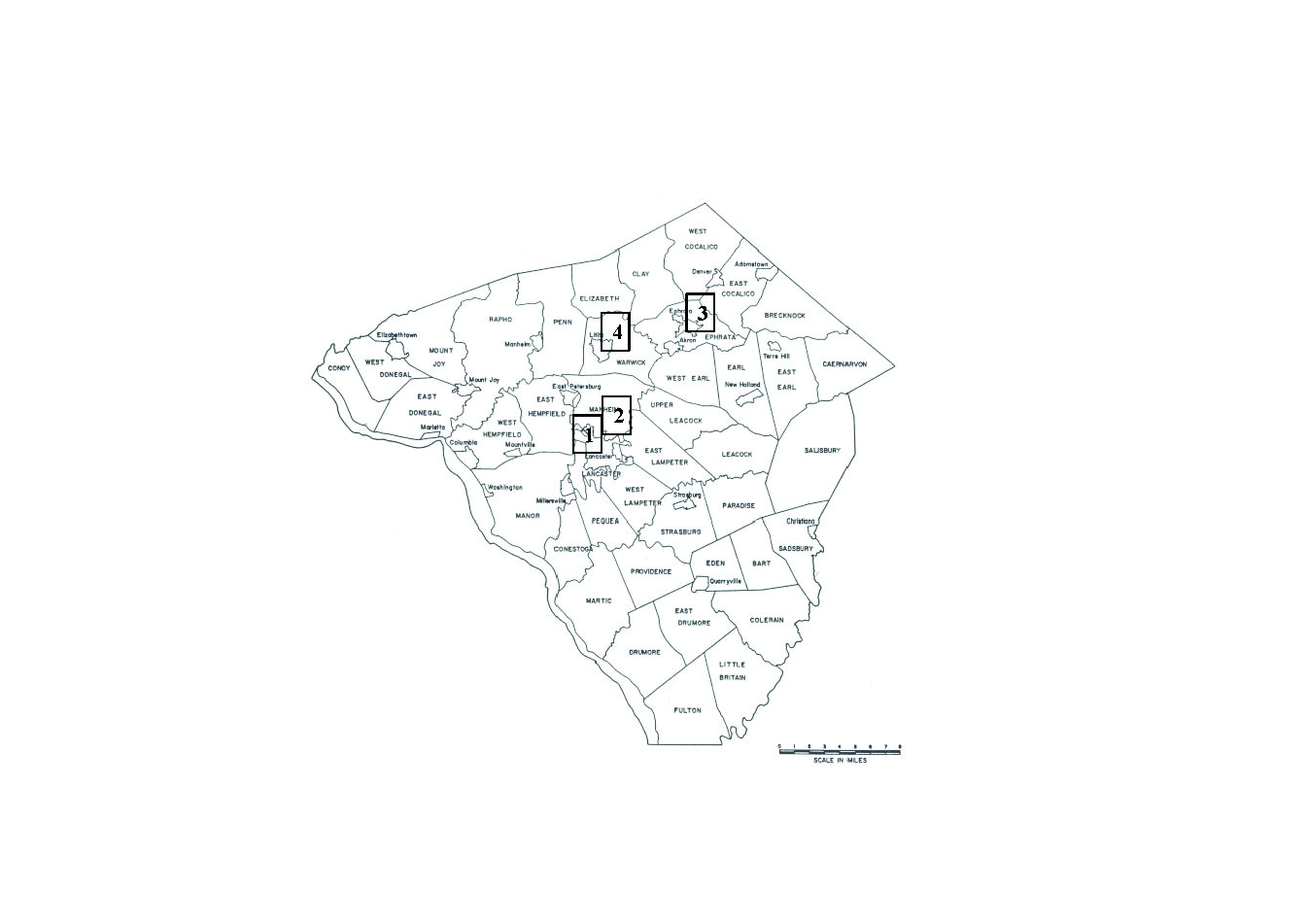

Supplement: Figure S1 — Lancaster County, including locations of Lancaster General Community Hospital (1), Lancaster Regional Medical Center (2), Ephrata Community Hospital (3), and Ephrata Community Hospital (4). From http://www.lancasterhistory.org/images/stories/lancaster_county_history/map_townships.jpg. (TIF) [file pone.0051560.s001.tif]

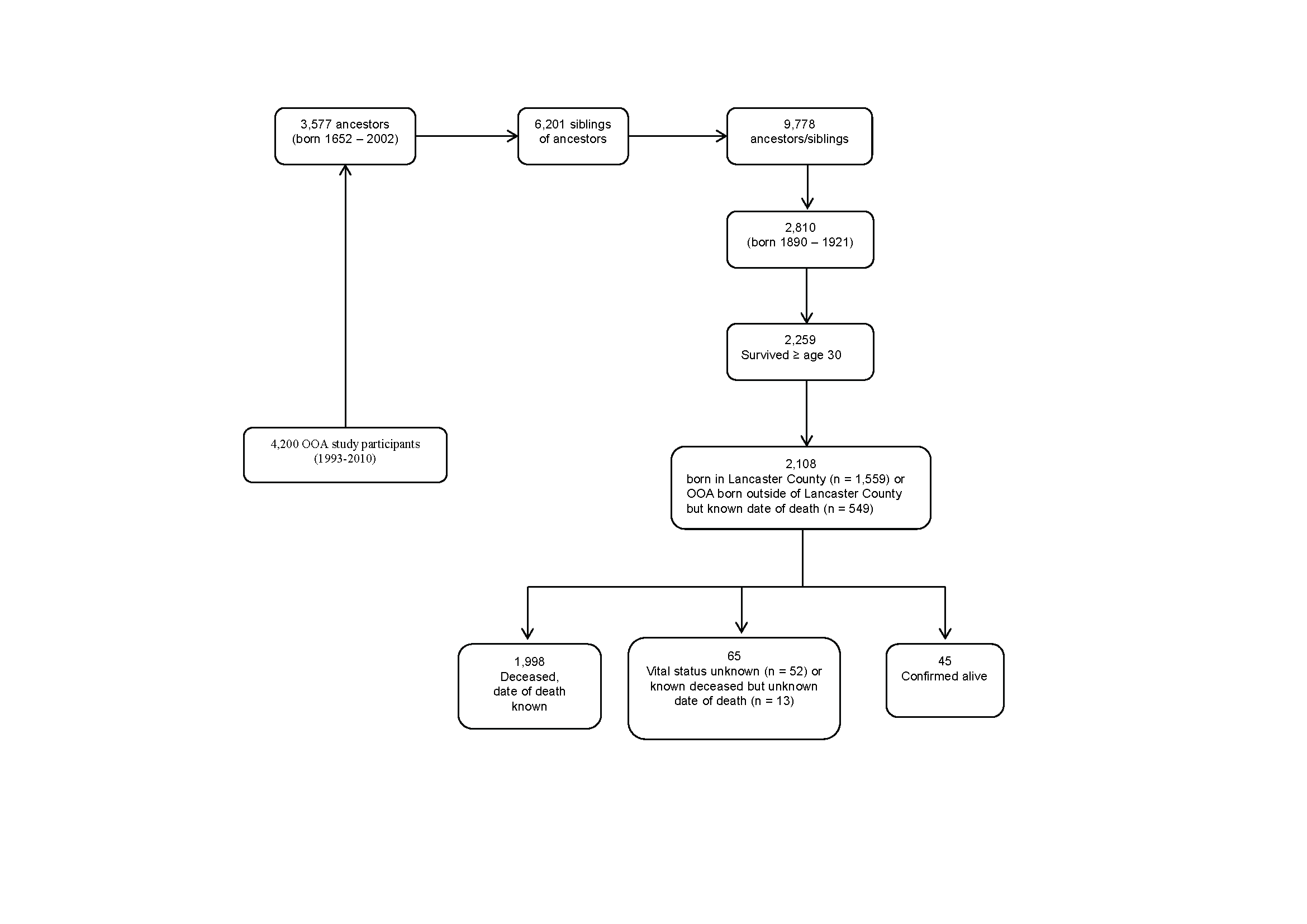

Supplement: Figure S2 — Construction of the OOA mortality cohort. (TIF) [file pone.0051560.s002.tif]
