# Supplementary material for: Living the Good Life? Mortality and Hospital Utilization Patterns in the Old Order Amish
Source: PLoS One. 2012 Dec 19;7(12):e51560. doi: 10.1371/journal.pone.0051560 (PMC3526600; doi:10.1371/journal.pone.0051560)
Supplement: Table S1 — Age and sex distribution of the Lancaster County Old Order Amish population as of July 1st 2002 * (percentages in parentheses). (DOCX) [file pone.0051560.s003.docx]

| Supplementary Table 1. Age and sex distribution of the Lancaster County Old Order Amish population as of July 1^st^ 2002 * (percentages in parentheses) | | | |
| --- | --- | --- | --- |
| Age | Male | Female | Total |
| 0-24 years | 7,229 (65.8) | 6,745 (64.3) | 13,974 (65.1) |
| 25-44 years | 2,160 (19.7) | 2,024 (19.3) | 4,184 (19.5) |
| 45-64 years | 1,179 (10.7) | 1,188 (11.3) | 2,367 (11.0) |
| 65+ years | 414 (3.8) | 537 (5.1) | 950 (4.4) |
| Total N (%) | 10,982 (100) | 10,494 (100) | 21,476 (100) |
| * extrapolated from the 2002 Church Directory of the Lancaster County Amish (see text for details) | | | |
