# Supplementary material for: Living the Good Life? Mortality and Hospital Utilization Patterns in the Old Order Amish
Source: PLoS One. 2012 Dec 19;7(12):e51560. doi: 10.1371/journal.pone.0051560 (PMC3526600; doi:10.1371/journal.pone.0051560)
Supplement: Table S2 — Three-year rates of first-listed hospital discharges (per 10,000) among Old Order Amish residing in Lancaster County, Pennsylvania, 2002–2004. (DOCX) [file pone.0051560.s004.docx]

| Supplementary Table 2: Three-year rates of first-listed hospital discharges (per 10,000) among Old Order Amish residing in Lancaster County, Pennsylvania, 2002-2004 | | | | | | | | |
| --- | --- | --- | --- | --- | --- | --- | --- | --- |
|  | **Men** | | | | **Women** | | | |
| **Diagnosis** | All ages | 25-44 yrs. | 45-64 yrs. | 65+ yrs. | All ages | 25-44 yrs. | 45-64 yrs. | 65+ yrs. |
| Infectious and parasitic diseases (001–139) | 2.7 (1)† | 4.6 (1) | 0.0 (0) | 0.0 (0) | 2.7 (1) | 0.0 (0) | 0.0 (0) | 18.6 (1) |
| Neoplasms (140–239) | 48.0 (18) | 9.3 (2) | 84.8 (10) | 144.9 (6) | 29.3 (11) | 9.9 (2) | 58.9 (7) | 37.2 (2) |
| Endocrine, nutritional and metabolic diseases, and immunity disorders (240–279) | 13.3 (5) | 4.6 (1) | 17.0 (2) | 48.3 (2) | 5.3 (2) | 0.0 (0) | 0.0 (0) | 37.2 (2) |
| Diseases of the blood and blood-forming organs (280–289) | 10.7 (4) | 0.0 (0) | 0.0 (0) | 96.6 (4) | 0.0 (0) | 0.0 (0) | 0.0 (0) | 0.0 (0) |
| Mental disorders (290–319) | 21.3 (8) | 23.2 (5) | 8.5 (1) | 48.3 (2) | 5.3 (2) | 4.9 (1) | 8.4 (1) | 0.0 (0) |
| Diseases of the nervous system and sense organs (320–389) | 2.7 (1) | 4.6 (1) | 0.0 (0) | 0.0 (0) | 0.0 (0) | 0.0 (0) | 0.0 (0) | 0.0 (0) |
| Diseases of the circulatory system (390–459) | 111.9 (42) | 4.6 (1) | 135.7 (16) | 603.9 (25) | 104.0 (39) | 19.8 (4) | 50.5 (6) | 540.0 (29) |
| Diseases of the respiratory system (460–519) | 66.6 (25) | 13.9 (3) | 67.9 (8) | 338.2 (14) | 32.0 (12) | 4.9 (1) | 25.3 (3) | 149.0 (8) |
| Diseases of the digestive system (520–579) | 69.3 (26) | 41.7 (9) | 59.4 (7) | 241.6 (10) | 50.7 (19) | 24.7 (5) | 58.9 (7) | 130.4 (7) |
| Diseases of the genitourinary system (580–629) | 13.5 (5) | 0.0 (0) | 8.5 (1) | 96.6 (4) | 40.0 (15) | 14.8 (3) | 58.9 (7) | 93.1 (5) |
| Complications of pregnancy, childbirth, and the puerperium (630–679) | 0.0 (0) | 0.0 (0) | 0.0 (0) | 0.0 (0) | 253.4 (95) | 459.5 (93) | 16.8 (2) | 0.0 (0) |
| Diseases of the skin and subcutaneous tissue (680–709) | 5.3 (2) | 4.6 (1) | 8.5 (1) | 0.0 (0) | 10.7 (4) | 0.0 (0) | 16.8 (2) | 37.2 (2) |
| Diseases of the musculoskeletal system and connective tissue (710–739) | 61.3 (23) | 18.5 (4) | 93.3 (11) | 193.2 (8) | 45.4 (17) | 14.8 (3) | 50.5 (6) | 149.0 (8) |
| Congenital anomalies (740–759) | 5.3 (2) | 4.6 (1) | 8.5 (1) | 0.0 (0) | 2.7 (1) | 0.0 (0) | 8.4 (1) | 0.0 (0) |
| Certain conditions originating in the perinatal period (760–779) | 0.0 (0) | 0.0 (0) | 0.0 (0) | 0.0 (0) | 0.0 (0) | 0.0 (0) | 0.0 (0) | 0.0 (0) |
| Symptoms, signs, and ill-defined conditions (780–799)* | 42.6 (16) | 13.9 (3) | 76.3 (9) | 96.6 (4) | 13.3 (5) | 9.9 (2) | 8.4 (1) | 37.2 (2) |
| Injury and poisoning (800–999) | 85.3 (32) | 37.0 (8) | 93.3 (11) | 314.0 (13) | 64.0 (24) | 29.6 (6) | 50.5 (6) | 223.5 (12) |
| Supplementary classification (V01–V91)** | 26.7(10) | 4.6 (1) | 76.3 (9) | 0.0 (0) | 2.7 (1) | 0.0 (0) | 8.4 (1) | 0.0 (0) |
| † Number of discharges in parentheses; * Symptoms = alteration of consciousness, hallucinations, syncope and collapse, convulsions, dizziness, sleep disturbances, fever, malaise and fatigue, hyperhidrosis and other general symptoms; ** Supplemental = potential health hazards related to different personal and family circumstances, and health services encountered for different reasons including birth. | | | | | | | | |
