# Supplementary material for: Living the Good Life? Mortality and Hospital Utilization Patterns in the Old Order Amish
Source: PLoS One. 2012 Dec 19;7(12):e51560. doi: 10.1371/journal.pone.0051560 (PMC3526600; doi:10.1371/journal.pone.0051560)
Supplement: Table S3 — Three-year rates of first-listed hospital discharges (per 10,000), Caucasians from the NHDS, 2002–2004. (DOCX) [file pone.0051560.s005.docx]

Supplementary Table 3: Three-year rates of first-listed hospital discharges (per 10,000), Caucasians from the NHDS, 2002-2004.

|  | **Men** | | | | **Women** | | | |
| --- | --- | --- | --- | --- | --- | --- | --- | --- |
| **Diagnosis** | **All ages** | **25-44 yrs.** | **45-64 yrs.** | **65+ yrs.** | **All ages** | **25-44 yrs.** | **45-64 yrs.** | **65+ yrs.** |
| Infectious and parasitic diseases (001–139) | 74.4  (553,775)† | 35.4  (119,869) | 52.0  (142,699) | 222.7  (291,207) | 90.3  (720,706) | 31.9  (106,021) | 59.1  (167,353) | 245.2  (447,332) |
| Neoplasms (140–239) | 158.5  (1,179,228) | 23.0  (77,841) | 154.9  (424,796) | 517.4  (676,591) | 219.0  (1,747,809) | 105.8  (351,462) | 231.7  (656,626) | 405.5  (739,721) |
| Endocrine, nutritional and metabolic diseases, and immunity disorders (240–279) | 134.8  (1,002,594) | 57.7  (195,575) | 124.5  (341,314) | 356.1  (465,705) | 193.7  (1,546,012) | 82.1  (272,602) | 154.7  (438,309) | 457.8  (835,101) |
| Diseases of the blood and blood-forming organs (280–289) | 30.3  (225,573) | 6.1  (20,726) | 24.2  (66,442) | 105.8  (138,405) | 45.1  (360,019) | 11.0  (36,484) | 31.6  (89,469) | 128.3  (234,066) |
| Mental disorders (290–319) | 211.6  (1,573,943) | 242.8  (823,003) | 203.1  (556,815) | 148.5  (194,125) | 204.5 (1,631,641) | 227.4  (755,208) | 192.3  (544,954) | 181.7  (331,479) |
| Diseases of the nervous system and sense organs (320–389) | 43.7  (324,890) | 19.9  (67,563) | 34.7  (95,228) | 124.0  (162,099) | 60.5  (482,438) | 35.9  (119,280) | 46.5  (131,724) | 126.9  (231,434) |
| Diseases of the circulatory system (390–459) | 819.9  (6,098,926) | 116.9  (396,193) | 735.7 (2,017,324) | 2818.3  (3,685,409) | 739.7  (5,902,575) | 74.0  (245,763) | 464.5  (1,316,282) | 2379.2  (4,340,530) |
| Diseases of the respiratory system (460–519) | 321.3  (2,390,067) | 59.9  (203,114) | 218.5  (599,149) | 1214.2  (1,587,804) | 384.2  (3,065,856) | 82.9  (275,339) | 262.7  (744,581) | 1121.5  (2,045,936) |
| Diseases of the digestive system (520–579) | 328.2  (2,441,634) | 159.2  (539,682) | 316.8  (868,677) | 790.2  (1,033,275) | 408.8  (3,262,112) | 184.8  (613,988) | 336.8  (954,535) | 928.3  (1,693,589) |
| Diseases of the genitourinary system (580–629) | 133.0  (989,433) | 35.6  (120,802) | 98.6  (270,424) | 457.5  (598,207) | 279.6  (2,231,461) | 228.9  (760,329) | 217.9  (617,579) | 467.9  (853,553) |
| Complications of pregnancy, childbirth, and the puerperium (630–679) | 0.0 | 0.0 | 0.0 | 0.0 | 61.0  (486,982) | 146.2  (485,731) | 0.4  (1,251) | 0.0 |
| Diseases of the skin and subcutaneous tissue (680–709) | 66.7  (496,449) | 41.9  (142,042) | 67.5  (185,117) | 129.5  (169,290) | 63.2  (504,741) | 31.4  (104,327) | 48.1  (136,367) | 144.7  (264,047) |
| Diseases of the musculoskeletal system and connective tissue (710–739) | 199.7  (1,485,420) | 82.5  (279,718) | 215.2  (590,077) | 470.8  (615,625) | 250.0  (1,995,011) | 70.8  (235,159) | 237.2  (672,144) | 596.2  (1,087,708) |
| Congenital anomalies (740–759) | 5.0  (36,830) | 5.8  (19,691) | 4.1  (11,320) | 4.4  (5,819) | 7.3  (58,102) | 7.1  (23,570) | 8.9  (25,245) | 5.1  (9,287) |
| Certain conditions originating in the perinatal period (760–779) | 0.4  (3,220) | 1.0  (3,220) | 0.0 | 0.0 | 0.6  (4,893) | 1.4  (4,564) | 0.0 | 0.2  (329) |
| Symptoms, signs, and ill-defined conditions (780–799)* | 20.8  (154,750) | 14.4 (48,757) | 23.3  (63,785) | 32.3  (42,208) | 24.0  (191,817) | 20.6  (68,468) | 20.0  (56,761) | 36.5  (66,588) |
| Injury and poisoning (800–999) | 262.0  (1,949,240) | 162.9  (552,060) | 234.8  (643,782) | 576.1  (753,398) | 298.7  (2,383,600) | 117.3  (389,752) | 202.2  (573,160) | 778.7  (1,420,688) |
| Supplementary classification (V01–V91)** | 89.1  (662,808) | 21.4  (72,677) | 72.5  (198,834) | 299.2  (391,297) | 663.7  (5,296,479) | 1330.6  (4,419,896) | 80.1  (227,029) | 356.0  (649,554) |
| † Number of discharges in parentheses; * Symptoms = alteration of consciousness, hallucinations, syncope and collapse, convulsions, dizziness, sleep disturbances, fever, malaise and fatigue, hyperhidrosis and other general symptoms; ** Supplemental = potential health hazards related to different personal and family circumstances, and health services encountered for different reasons including birth. | | | | | | | | |
