# Supplementary material for: Living the Good Life? Mortality and Hospital Utilization Patterns in the Old Order Amish
Source: PLoS One. 2012 Dec 19;7(12):e51560. doi: 10.1371/journal.pone.0051560 (PMC3526600; doi:10.1371/journal.pone.0051560)
Supplement: Table S4 — Three-year rates of any-listed hospital discharges (per 10,000) among Old Order Amish residing in Lancaster County, Pennsylvania, 2002–2004. (DOCX) [file pone.0051560.s006.docx]

| Supplementary Table 4: Three-year rates of any-listed hospital discharges (per 10,000) among Old Order Amish residing in Lancaster County, Pennsylvania, 2002-2004 | | | | | | | | | |
| --- | --- | --- | --- | --- | --- | --- | --- | --- | --- |
|  | Men | | | | Women | | | |  |
| **Diagnosis** | All ages | 25-44 yrs. | 45-64 yrs. | 65+ yrs. | All ages | 25-44 yrs. | 45-64 yrs. | 65+ yrs. |  |
| Infectious and parasitic diseases (001–139) | 77.3 (29)† | 27.8 (6) | 110.3 (13) | 241.5 (10) | 37.3 (14) | 14.8 (3) | 8.4 (1) | 186.2 (10) |  |
| Neoplasms (140–239) | 114.6 (43) | 13.9 (3) | 229.0 (27) | 314.0 (13) | 85.4 (32) | 14.8 (3) | 193.6 (23) | 111.7 (6) |  |
| Endocrine, nutritional and metabolic diseases, and immunity disorders (240–279) | 285.1 (107) | 50.9 (11) | 305.3 (36) | 1449.3 (60) | 266.7 (100) | 64.2 (13) | 218.9 (26) | 1135.9 (61) |  |
| Diseases of the blood and blood-forming organs (280–289) | 151.9 (57) | 23.1 (5) | 195.1 (23) | 700.5 (29) | 93.4 (35) | 39.5 (8) | 92.6 (11) | 298.0 (16) |  |
| Mental disorders (290–319) | 111.9 (42) | 55.6 (12) | 127.2 (15) | 362.3 (15) | 37.3 (14) | 9.9 (2) | 25.3 (3) | 167.6 (9) |  |
| Diseases of the nervous system and sense organs (320–389) | 53.3 (20) | 18.5 (4) | 42.4 (5) | 265.7 (11) | 40.0 (15) | 9.9 (2) | 33.7 (4) | 167.6 (9) |  |
| Diseases of the circulatory system (390–459) | 610.2 (229) | 27.8 (6) | 729.4 (86) | 3309.2 (137) | 546.8 (205) | 59.3 (12) | 319.9 (38) | 2886.4 (155) |  |
| Diseases of the respiratory system (460–519) | 226.5 (85) | 55.6 (12) | 262.9 (31) | 1014.5 (42) | 98.7 (37) | 14.8 (3) | 84.2 (10) | 446.9 (24) |  |
| Diseases of the digestive system (520–579) | 170.5 (64) | 69.4 (15) | 144.2 (17) | 772.9 (32) | 128.0 (48) | 69.2 (14) | 126.3 (15) | 353.8 (19) |  |
| Diseases of the genitourinary system (580–629) | 79.0 (30) | 13.9 (3) | 67.9 (8) | 458.9 (19) | 170.7 (64) | 74.1 (15) | 252.5 (30) | 353.8 (19) |  |
| Complications of pregnancy, childbirth, and the puerperium (630–679) | 0 (0) | 0 (0) | 0 (0) | 0 (0) | 634.8 (238) | 1126.5 (228) | 84.2 (10) | 0 (0) |  |
| Diseases of the skin and subcutaneous tissue (680–709) | 16.0 (6) | 9.3 (2) | 25.4 (3) | 24.2 (1) | 32.0 (12) | 4.9 (1) | 58.9 (7) | 74.5 (4) |  |
| Diseases of the musculoskeletal system and connective tissue (710–739) | 143.9 (54) | 37.0 (8) | 271.4 (32) | 338.2 (14) | 128.0 (48) | 19.8 (4) | 151.5 (18) | 484.2 (26) |  |
| Congenital anomalies (740–759) | 8.0 (3) | 4.6 (1) | 17.0 (2) | 0 (0) | 8.0 (3) | 9.9 (2) | 8.4 (1) | 0 (0) |  |
| Certain conditions originating in the perinatal period (760–779) | 0 (0) | 0 (0) | 0 (0) | 0 (0) | 0 (0) | 0 (0) | 0 (0) | 0 (0) |  |
| Symptoms, signs, and ill-defined conditions (780–799)* | 133.2 (50) | 32.4 (7) | 220.5 (26) | 410.6 (17) | 125.4 (47) | 44.5 (9) | 84.2 (10) | 521.4 (28) |  |
| Injury and poisoning (800–999) | 173.2 (65) | 88.0 (19) | 237.5 (28) | 434.8 (18) | 96.0 (36) | 34.6 (7) | 92.6 (11) | 335.2 (18) |  |
| Supplementary classification (V01–V91)** | 274.4 (103) | 64.8 (14) | 364.7 (43) | 1111.1 (46) | 397.4 (149) | 563.2 (114) | 75.8 (9) | 484.2 (26) |  |
| † Number of discharges in parentheses; * Symptoms = alteration of consciousness, hallucinations, syncope and collapse, convulsions, dizziness, sleep disturbances, fever, malaise and fatigue, hyperhidrosis and other general symptoms; ** Supplemental = potential health hazards related to different personal and family circumstances, and health services encountered for different reasons including birth. | | | | | | | | |  |
