# Supplementary material for: Living the Good Life? Mortality and Hospital Utilization Patterns in the Old Order Amish
Source: PLoS One. 2012 Dec 19;7(12):e51560. doi: 10.1371/journal.pone.0051560 (PMC3526600; doi:10.1371/journal.pone.0051560)
Supplement: Table S5 — Three-year rates of any-listed hospital discharges (per 10,000), Caucasians from the NHDS, 2002–2004. (DOCX) [file pone.0051560.s007.docx]

| Supplementary Table 5: Three-year rates of any-listed hospital discharges (per 10,000), Caucasians from the NHDS, 2002-2004. | | | | | | | | |
| --- | --- | --- | --- | --- | --- | --- | --- | --- |
|  | Men | | | | Women | | | |
| **Diagnosis** | All ages | 25-44 yrs. | 45-64 yrs. | 65+ yrs. | All ages | 25-44 yrs. | 45-64 yrs. | 65+ yrs. |
| Infectious and parasitic diseases (001–139) | 353.6  (2,630,183)† | 163.6  (554,515) | 315.5  (865,149) | 925.7  (1,210,519) | 426.2  (3,401,218) | 172.9  (574,334) | 288.8  (818,328) | 1101.0  (2,008,556) |
| Neoplasms (140–239) | 465.3  (3,461,416) | 61.3  (207,824) | 405.0  (1,110,361) | 1639.0  (2,143,231) | 573.8  (4,578,973) | 231.7  (769,705) | 565.6  (1,602,950) | 1209.4  (2,206,318) |
| Endocrine, nutritional and metabolic diseases, and immunity disorders (240–279) | 1807.8  (13,447,813) | 400.9  (1,358,767) | 1715.0  (4,702,565) | 5648.6  (7,386,481) | 2359.5  (18,829,194) | 583.0  (1,936,522) | 1791.0  (5,075,602) | 6477.4  (11,817,070) |
| Diseases of the blood and blood-forming organs (280–289) | 413.2  (3,074,074) | 88.9  (301,170) | 327.0  (896,747) | 1434.7  (1,876,157) | 581.4  (4,639,663) | 214.3  (711,932) | 349.9  (991,601) | 1609.4  (2,936,130) |
| Mental disorders (290–319) | 1107.5  (8,238,534) | 909.9  (3,083,934) | 1123.2  (3,079,711) | 1586.7  (2,074,889) | 1228.8  (9,805,762) | 936.3  (3,110,113) | 1027.5  (2,912,004) | 2074.0  (3,783,645) |
| Diseases of the nervous system and sense organs (320–389) | 352.1  (2,618,983) | 110.4  (374,309) | 268.7  (736,772) | 1153.1  (1,507,902) | 458.4  (3,657,814) | 163.6  (543,420) | 289.3  (819,987) | 1257.7  (2,294,407) |
| Diseases of the Circulatory System (390–459) | 4100.5  (30,503,112) | 451.1  (1,529,040) | 3154.0  (8,648,203) | 15543.7  (20,325,869) | 4153.8  (33,147,832) | 395.5  (1,313,625) | 2275.5  (6,448,615) | 13914.8  (25,385,592) |
| Diseases of the respiratory system (460–519) | 1190.1  (8,852,738) | 216.5  (733,631) | 870.8  (2,387,587) | 4383.0  (5,731,520) | 1339.8  (10,691,467) | 315.3  (1,047,347) | 902.1  (2,556,433) | 3885.0  (7,087,687) |
| Diseases of the digestive system (520–579) | 1029.5  (7,658,698) | 402.9  (1,365,566) | 992.7  (2,721,882) | 2731.0  (3,571,250) | 1350.0  (10,773,254) | 521.4  (1,731,877) | 1093.3  (3,098,390) | 3257.6  (5,942,987) |
| Diseases of the genitourinary system (580–629) | 704.1  (5,237,988) | 118.8  (402,590) | 439.7  (1,205,634) | 2775.8  (3,629,764) | 1164.5  (9,292,940) | 798.4  (2,652,225) | 860.3  (2,438,135) | 2303.6  (4,202,580) |
| Complications of pregnancy, childbirth, and the puerperium (630–679) | 0  (0) | 0  (0) | 0  (0) | 0  (0) | 1331.5  (10,625,500) | 3,186.7  (10,585,455) | 14.1  (40,045) | 0  (0) |
| Diseases of the skin and subcutaneous tissue (680–709) | 241.9  (1,799,561) | 99.1  (335,853) | 224.3  (614,953) | 649.1  (848,755) | 251.3  (2,005,263) | 90.9  (301,919) | 185.3  (525,051) | 645.9  (1,178,293) |
| Diseases of the musculoskeletal system and connective tissue (710–739) | 495.6  (3,686,625) | 19.2  (65,156) | 550.5  (1,509,426) | 1615.1  (2,112,043) | 936.1  (7,470,508) | 33.2  (110,338) | 739.1  (2,094,690) | 2886.2  (5,265,480) |
| Congenital anomalies (740–759) | 20.7  (153,616) | 5.8  (19,691) | 29.2  (79,962) | 41.3  (53,963) | 24.1  (192,666) | 7.1  (23,570) | 34.0  (96,441) | 39.8  (72,655) |
| Certain conditions originating in the perinatal period (760–779) | 1.7  (12,925) | 2.3  (7,919) | 0.5  (1,504) | 2.7  (3,502) | 1.6  (13,048) | 3.2  (10,689) | 0.1  (358) | 1.1  (2,001) |
| Symptoms, signs, and ill-defined conditions (780–799)* | 747.8  (5,563,002) | 303.4  (1,028,439) | 701.1  (1,922,298) | 1997.7  (2,612,265) | 927.3  (7,399,876) | 419.2  (1,392,512) | 742.7  (2,104,733) | 2139.2  (3,902,631) |
| Injury and poisoning (800–999) | 656.5  (4,883,449) | 408.5  (1,384,426) | 573.1  (1,571,520) | 1474.0  (1,927,503) | 652.6  (5,207,697) | 282.2  (937,321) | 454.4  (1,287,789) | 1634.9  (2,982,587) |
| Supplementary classification (V01–V91)** | 928.7  (6,908,315) | 226.0  (765,900) | 766.4  (2,101,463) | 3090.2  (4,040,952) | 1626.4  (12,979,128) | 1,910.1  (6,344,948) | 693.2  (1,964,533) | 2559.6  (4,669,647) |
| † Number of discharges in parentheses; * Symptoms = alteration of consciousness, hallucinations, syncope and collapse, convulsions, dizziness, sleep disturbances, fever, malaise and fatigue, hyperhidrosis and other general symptoms; ** Supplemental = potential health hazards related to different personal and family circumstances, and health services encountered for different reasons including birth. | | | | | | | | |
