# Supplementary material for: Living the Good Life? Mortality and Hospital Utilization Patterns in the Old Order Amish
Source: PLoS One. 2012 Dec 19;7(12):e51560. doi: 10.1371/journal.pone.0051560 (PMC3526600; doi:10.1371/journal.pone.0051560)
Supplement: Table S6 — Standardized ratios* comparing hospital discharge rates for any-listed diagnostic procedures between Old Order Amish and non-Amish whites, 2002–2004. Non-Amish white rates from the NHDS. (DOCX) [file pone.0051560.s008.docx]

| Supplementary Table 6. Standardized ratios* comparing hospital discharge rates for any-listed diagnostic procedures between Old Order Amish and non-Amish whites, 2002-2004. Non-Amish white rates from the NHDS. | | | |
| --- | --- | --- | --- |
| **Procedures on the following systems** | Males | Females | Total |
|  | O/E ± SE (O/E) | O/E ± SE (O/E) | O/E ± SE (O/E) |
| **Nervous** | 0.94 ± 0.17 | 0.13 ± 0.06 | 0.48 ± 0.08 |
| **Endocrine** | 0.00 | 0.00 | 0.00 |
| **Eye** | 0.00 | 0.00 | 0.00 |
| **Ear** | 0.00 | 0.00 | 0.00 |
| **Nose, throat** | 0.30 ± 0.17 | 0.00 | 0.19 ± 0.11 |
| **Respiratory** | 0.35 ± 0.10 | 0.23 ± 0.09 | 0.30 ± 0.07 |
| **Cardiovascular** | 0.27 ± 0.03 | 0.24 ± 0.04 | 0.26 ± 0.02 |
| **Hemic** | 0.18 ± 0.13 | 0.08 ± 0.08 | 0.13 ± 0.08 |
| **Digestive** | 0.31 ± 0.04 | 0.19 ± 0.03 | 0.24 ± 0.02 |
| **Urinary** | 0.30 ± 0.10 | 0.24 ± 0.08 | 0.27 ± 0.06 |
| **Male genital** | 0.00 | - | 0.00 |
| **Female genital** | - | 0.23 ± 0.04 | 0.23 ± 0.04 |
| **Obstetric** | - | 0.33 ± 0.03 | 0.33 ± 0.03 |
| **Musculoskeletal** | 0.36 ± 0.05 | 0.32 ± 0.05 | 0.34 ± 0.04 |
| **Integumentary** | 0.19 ± 0.07 | 0.33 ± 0.08 | 0.27 ± 0.05 |
| **Miscellaneous** | 0.21 ± 0.02 | 0.16 ± 0.02 | 0.18 ± 0.02 |
| **Diagnostic procedures** | 0.18 ± 0.03 | 0.17 ± 0.03 | 0.18 ± 0.02 |
| * Standardized ratios computed using the indirect age adjustment method (see text for details) | | | |
