# Supplementary material for: Living the Good Life? Mortality and Hospital Utilization Patterns in the Old Order Amish
Source: PLoS One. 2012 Dec 19;7(12):e51560. doi: 10.1371/journal.pone.0051560 (PMC3526600; doi:10.1371/journal.pone.0051560)
Supplement: Information S1 — (DOCX) [file pone.0051560.s009.docx]

Supplementary Information

Living the Good Life? Mortality and Hospital Utilization Patterns in the Lancaster County Amish

1. Estimation of the census of the Lancaster County Old Order Amish (OOA)
2. Construction of Anabaptist Genealogy Database Version 5 (AGDB5)
3. Construction of the OOA Mortality Cohort
4. Comparison of severity of illness between OOA and US Caucasions
5. **Estimation of the census of the Lancaster County Old Order Amish (OOA)**

We estimated the population size of the Lancaster County Amish community using residence information obtained from the 2002 Church Directory of the Lancaster County Amish. [^1^](#_ENREF_1) This Directory is published by the Amish and is periodically updated. In 2002, the Amish Lancaster community included 141 distinct OOA church districts, of which 132 are situated in Lancaster County. Each district included 17 - 50 households (mean number of families per district = 30). [^2^](#_ENREF_2) The Church Directory provides for each district a listing of all household members and their birthdates. We sampled 30 of these districts, selected at random, (representing 23% of all districts) and enumerated the number of OOA individuals by age and sex, and then used these numbers to extrapolate the numbers of OOA in all 132 districts in Lancaster County. We used the Church Directory rather than AGDB5 for census estimation in order to base our estimate on the Lancaster County OOA only, since only this subgroup would reside in the encatchment zone of the four Lancaster County area hospitals.

1. **Construction of Anabaptist Genealogy Database Version 5 (AGDB5)**

Anabaptist Genealogy Database (AGDB) is a computer-searchable genealogy database that includes the Old Order Amish (OOA) of Lancaster County in Pennsylvania and other Anabaptist populations. There are multiple versions of AGDB [^3-7^](#_ENREF_3) constructed using various sources and updates. We parsed each source, resolved syntactic errors, identified duplicate individuals and adoptees, matched individuals across sources, and generated a set of result files as a computer-searchable database. The reason to distinguish adoptees is that users of AGDB are interested in biological parent-child relationships in which genes are inherited.

**Three Sources to Construct Multiple Versions of AGDB**

The initial source to construct AGDB was a computer file updating the *Fisher Family History* (FFH) book [^8^](#_ENREF_8) edited by Ms. Katie Beiler; the computer file was received in 1996. AGDB version 1 (AGDB1) [^3^](#_ENREF_3) completed in 1998 contains 55,636 individuals and 12,896 marriages. The *Amish and Amish Mennonite Genealogies* (AAMG) book [^9^](#_ENREF_9) was integrated into later versions of AGDB. AGDB version 2 (AGDB2) [^4^](#_ENREF_4) completed in 1999 contains 85,720 individuals and 26,154 marriages. We integrated a large computerized genealogy file containing many geography regions received from Mr. James C. Hostetler in 2000 and a small update of recent births, deaths and marriages in the Lancaster area received from Ms. Beiler in 1999 to make AGDB version 3 (AGDB3). AGDB3 was completed in 2001 and contains 295,125 individuals and 68,145 marriages. We then integrated the second edition of the large computerized genealogy file from Mr. Hostetler and another small update from Ms. Beiler to arrive at AGDB version 4 (AGDB4), [^7^](#_ENREF_7) which was completed in 2004 and contains 417,789 individuals and 102,341 marriages.

In 2009, we received a computer file of the new edition of the FFH book (FFH2009) [^10^](#_ENREF_10) edited by Ms. Beiler. In 2010, we received the third edition of the large computerized genealogy file (Hostetler2010) from Mr. Hostetler. We then took multiple steps to parse and merge all the above sources and updates to construct AGDB version 5 (AGDB5). Instead of re-parsing the AAMG book, we used the full AGDB4 to be merged with FFH2009 and Hostetler2010 to construct AGDB5.

**Parsing FFH2009 and Hostetler2010**

FFH2009 was obtained in Microsoft Word document files. We first converted the files into ASCII plain text files. Each record in FFH2009 contains information about a nuclear family. The record format in the 2009 edition (shown below) is similar but not identical to the format in the 1996 computer file. Therefore, we could do an initial parse of the book using a slightly modified version of the C language developed for the original FFH parsing task. [^8^](#_ENREF_8) An example record is as follows:

| *1. Christian Fisher b April 26, 1757, d Nov. 19, 1838, m Barbara Yoder (Yost Yoder) OOA. Children: Barbara (2), Magdalena (7230), Catharine (9597). m2 Susanna Souder, b Mar. 10, 1757, d Sept. 28, 1831 (Henry and Barbara Stauffer Sauder). Children: Mary (11066), Christian, Jr. (11067), Elizabeth (13433), Anna (15967), Susanna (19059), Esther, b May 10, 1798, d May 4, 1820, of consumption; (some personal dishes which had belonged to her are still in possession of some of the Fisher descendants); Benjamin (21474).* |
| --- |

We collected following pieces of information from each record in FFH2009:

- Fisher number or family number; such as the leading *1* in the above example.
- Name of family head; such as *Christian Fisher* in the above example.
- If the parents of the family head are known, the Fisher number of that family is recorded in a pair of parentheses. The above example indicates that parents of *Christian Fisher* are unknown in this source; therefore, he is a founder.
- Birth and death dates are appended to an individual with key words *b* and *d* respectively. For the above example, *Christian Fisher* was born on *April 26, 1757* and died on *Nov. 19, 1838*.
- List of spouses. Each spouse starts with key word *m*, *m2*, or the like, and follows by name and dates. If the spouse is a family head of another family, the Fisher number is recorded in a pair of parentheses. If the parents of the spouse are known, the Fisher number of that family or parent names are recorded in a pair of parentheses. For the above example, the first spouse is *Barbara Yoder*, and her father is *Yost Yoder*; and the second spouse is *Susanna Souder*, *Mar. 10, 1757 - Sept. 28, 1831*, and her parents are *Henry and Barbara Stauffer Sauder*.
- List of children of each spouse is appended by key word *Children*. Each child starts with name. If the child or the spouse of the child is a family head of another family, the Fisher number of that family is recorded in a pair of parentheses; otherwise known dates are appended to the name.
- Some useful information, such as places, OOA status or adoption, could be included in a record.

The parsed results of person information and family relationships were stored in two separate output files structured as person table and relationship table that could be loaded into a relational database [^3^](#_ENREF_3). The parser identified thousands of errors in the source file that we corrected manually followed by re-parsing. The errors could be within a single record (e.g., inconsistent punctuation or an invalid date) or across records (e.g., two different spellings of an individual’s first name or a record number given in parentheses where the individual appears as a child does not match the record where the individual appears as a family head or spouse).

Hostetler2010 was obtained in a large GEDCOM-formatted file; GEDCOM is the standard format used by genealogists. We re-used a parser program written in the Perl programming language (originally developed for earlier versions of Mr. Hostetler’s file) to parse GEDCOM format. As for FFH, the parsing of Hostetler2010 outputs person table and relationship table into two separate output files, structured as relational tables. A third output file as ID table keeps the correspondence between the individual identifiers that start with *I* in GEDCOM and consecutive numeric IDs *1, 2, 3, …* assigned by the parser. Example individual and family records are as follows:

| *0 @I6378@ INDI*  *1 NAME Christian /Fisher/*  *1 SEX M*  *1 BIRT*  *2 DATE 26 APR 1757*  *2 PLAC PA*  *1 DEAT*  *2 DATE 19 NOV 1838*  *2 PLAC Lancaster Co., PA*  *1 NOTE Gingerich-Kreider FH13*  *1 FAMS @F1415@*  *1 FAMS @F3075@*  *1 FAMC @F1399@* | *0 @F1415@ FAM*  *1 HUSB @I6378@*  *1 WIFE @I2431@*  *1 CHIL @I7657@*  *1 CHIL @I7658@*  *1 CHIL @I7659@*  *1 MARR*  *2 PLAC PA* | *0 @F3075@ FAM*  *1 HUSB @I6378@*  *1 WIFE @I7660@*  *1 CHIL @I7661@*  *1 CHIL @I7662@*  *1 CHIL @I7663@*  *1 CHIL @I7664@*  *1 CHIL @I7665@*  *1 CHIL @I7666@*  *1 CHIL @I7667@*  *1 MARR*  *2 PLAC PA* |
| --- | --- | --- |

The column on the left is the individual record for the family head of the example from FFH2009. Two marriages in the FFH2009 example are recorded as two family records in the second and the third columns.

**Identifying Family Name, Gender and Adoption in Each Source**

Identifying family name and gender in the GEDCOM format should be straightforward. However due to human errors, we corrected hundreds of individual records (especially incorrect gender assignments) in Hostetler2010. Family heads and spouses in FFH2009 are recorded with full name. We parsed the last word excluding name suffix as family name for a given individual. To determine gender in FFH2009, we used statistics from AGDB4 to assist in the gender assignment, but there are hundreds of unmarried children to whom we did not assign either gender in AGDB5. All married individuals are assigned a gender.

To identify adoption relationships, we parsed key words *adopted*, *adopting*, *foster*, and symbols *(a)*, *(f)* in FFH 2009 and key words *adopted*, *adoption*, *adoptive*, *adopt*, and *foster* in Hostetler2010. We output adopting parents of the adoptees if they are known. We collected adoptees for FFH2009 and Hostetler2010 in two separate files as adoption tables. There are 358 adoptees in FFH2009 and 2,059 adoptees in Hostetler2010. We matched these against smaller lists of adoptees from AGDB4.

**Resolving Duplicates in Each Source**

To clean up and simplify each source, we wrote Ruby programs to identify individuals who seem to appear more than once within a source. The initial step to identify duplicates was to verify names and known birth and death dates. We allowed partial matches on names and exact matches on dates. We followed marriage links to verify whether the spouse was a duplicate. We then followed parent-child relationships to verify whether the parents or any child was a duplicate. We applied the transitive closure and recursively coalesced duplicates until no further duplicates were found. All duplicate pairs were kept in a separate file as duplicates table for each source. There are 39 duplicate pairs in FFH2009 and 89 duplicate pairs in Hostetler2010.

For the sake of backward compatibility to AGDB4 and compatibility across multiple sources, all duplicates are kept in person tables. For the sake of consistency in constructing pedigrees, only one copy of each duplicate appears in the relationship table.

**Matching Individuals across Sources**

We implemented Ruby programs to perform pair-wise matching of individuals among FFH2009, Hostetler2010 and AGDB4. Similar to identifying duplicates within each source, the initial matching between two sources allowed partial matches on names and exact matches on birth and death dates. We then extended matching along spouse and parent-child relationships. For example, after 52 rounds of matching, the comparison of FFH2009 and Hostetler2010 converged with 63,097 and 63,118 individuals respectively. The two cardinalities were not equal because of different numbers of duplicates between two sources, allowing one individual in one source to match two individuals in the other source. The matching between FFH2009 and AGDB4 converged with 74,327 and 74,357 matched individuals respectively. The matching between Hostetler2010 and AGDB4 converged with 393,145 and 393,144 matched individuals respectively.

Various Perl programs were implemented to check the automated matching and add pairs that were hard to find automatically. For example, Mr. Hostetler’s file annotates thousands of individuals as being in either the FFH or AAMG source. We identified the individuals with those annotations and checked either that they were matched or that Mr. Hostetler’s annotation did not agree with the claimed source.

**Determining Unique IDs and Merging Three Sources**

After identifying duplicates and updating matches accordingly, we wrote Perl programs and Shell scripts to merge FFH2009 and Hostetler2010 then AGDB4. We assigned consecutive numeric IDs to individuals in each source and made an ID correspondence table across three sources. We then removed adoptees as children from the relationship tables. To choose name, dates and places from multiple sources that disagree, we generally favored the record from FFH2009, then Hostetler2010 and finally AGDB4. For the purposes of the mortality study, what matters most are the dates; therefore, Table 1 describes what rule we used for each of the four possible combinations of two or more sources having distinct dates for an individual. In a few cases where this rule gave an implausible date (e.g., death date occurring years before parenthood), we investigated manually and either chose a plausible date from a lower priority source or left the date as unknown. In cases of incomplete name and dates from multiple sources, we chose the more complete record. We instantiated the AGDB5 database on SYBASE and loaded person table, relationship table, adoption table, duplicates table, ID table and ID correspondence table into the AGDB5 database. Finally, we tested AGDB5 using PedHunter 2.0. [^7^](#_ENREF_7)

**Table 1. If the same individual had distinct birth dates or death dates in multiple sources, we used the following rules to determine which date to be used in AGDB5.**

| **FFH2009** | **Hostetler2010** | **AGDB4** |  | **AGDB5** |
| --- | --- | --- | --- | --- |
| X | X |  |  | FFH2009 |
| X |  | X |  | FFH2009 |
|  | X | X |  | Hostetler2010 |
| X | X | X |  | FFH2009 |

AGDB5 contains 539,822 individuals and 136,214 marriages. Additionally, we identified 2,317 adoptees and 419 duplicates. The Venn diagram represented in Figure 1 summarizes the overlaps between different sources. As shown in the Venn diagram, the numbers of individuals from the three sources in AGDB5 are 106,651 from FFH++++ (contains the FFH book, three updates and FFH2009), 30,823 from AAMG, and 491,365 from Hostetler++ (contains three large computerized genealogy files in 2000, 2004, and 2010), among which only 1,125 individuals are commonly found in all three sources.

Figure 1. Venn diagram indicating overlaps between the three different sources that comprise AGDB5. To make the counts consistent, it is necessary to double count duplicates in whichever source has the duplicate. The FFH++++ years correspond to third edition published (1988), initial computer file (1996), update (1999), another update (2003), and fourth edition published (2009). The three years for Hostetler++ are the years in which we received updated versions of Mr. Hostetler’s file and these were included in AGDB3, AGDB4, and AGDB5 respectively.

| FFH++++ (**106,651**)  1988,1996,1999,  2003,2009  Hostetler++ (**491,365**) 2000,2004,2010  16,864  69,876  AAMG (**30,823**)  1986  1,125  12,807  27  35,623  403,500 |
| --- |
| **Figure 1. Venn diagram of overlaps among three sets of sources and updates in AGDB5**. FFH++++ contains 106,651 individuals in the FFH Third Edition published in 1988, the initial computer file in 1996, an update file in 1999, another update file in 2003, and the FFH Fourth Edition published in 2009. AAMG contains 30,823 individuals published in 1986. Hostetler++ contains 491,365 individuals in three versions of Mr. Hostetler’s large computerized genealogy file in 2000, 2004, and 2010. |

1. **Construction of the Amish mortality cohort**

We identified a cohort of OOA Amish individuals born between 1890 and 1921 whose vital status we ascertained. This Amish mortality cohort comprised ancestors and their siblings of the 4,200 OOA individuals who had participated in one or more of the population studies carried out in the Lancaster County community of OOA by our team of investigators at the University of Maryland from 1995-2010. [^11-14^](#_ENREF_11) We began with subjects we had studied to assure that they were practicing OOA living in Lancaster County. Identification of the relatives was made possible by accessing the Anabaptist Genealogy Database (AGDB), a computer-searchable genealogy database of the OOA and other Anabaptist populations dating back to the OOA immigration to the US that was generated and is managed by AAS and colleagues at the NIH. There are multiple versions of AGDB [^3-7^](#_ENREF_3) constructed using various sources and updates. The three major sources used to construct AGDB include the *Fisher Family History* (FFH) book, [^8^](#_ENREF_8)^,^[^10^](#_ENREF_10) The *Amish and Amish Mennonite Genealogies* (AAMG) book, [^9^](#_ENREF_9) and a large computerized genealogy file encompassing many geographic regions received from Mr. James C. Hostetler. We wrote computer programs to systematically match individuals across sources and resolve duplicates. The data for this analysis were compiled from AGDB5, which contains 539,822 individuals and 136,214 marriages.

From AGDB5, we identified all direct ancestors of our 4,200 OOA study participants (n = 3,577). Since by design, all ancestors of our current study participants would have had children and having children is associated with a longer lifespan than not having children, [^15^](#_ENREF_15)^,^[^16^](#_ENREF_16) we further included all siblings of these ancestors (n = 6,201 additional subjects, for a total of 9,778). We then restricted this set of ancestors and their siblings to those surviving until at least age 30 and born between 1890 and 1921 (n = 2,259); these restrictions were applied to assess relatively contemporary mortality rates, and for comparability with the Framingham cohort who enrolled in the Framingham Heart Study after age 28 and were born in approximately these same years. We further excluded subjects who had a recorded birth place outside of Lancaster County, but were missing a date of death (n = 151) because we had no mechanism to find out whether these 151 individuals died. At this stage, the OOA mortality cohort included 2,108 individuals of whom 121 were born in Lancaster County but had no date of death.

In the initial stage of mortality ascertainment, dates of death were obtained from the AGDB5 in almost all cases. Dates of death for approximately 40 individuals were obtained from the Swiss Anabaptist Genealogy Association (SAGA) web site. For the 121 individuals without a death date, the vital status was reviewed by our staff of research nurses and Amish liaisons. Additional field work was carried to update vital status by contacting families or contacts of the individual in question. From these efforts, we determined that 11 subjects had died with dates of death prior to September 1, 2011 and 45 subjects were confirmed to be alive as of January 1, 2011. An additional 13 subjects were reported by the AGDB5 or our liaisons as deceased but with unknown year of death; vital status was unknown in the remaining 52 subjects. These 65 subjects with unknown dates of death or unknown vital status were excluded from analysis. Mortality follow-up was thus complete on 96.9% (2,043/2,108) of the population. A summary detailing how we constructed the OOA mortality cohort is provided in Supplementary Figure S2.

1. **Comparison of severity of illness between OOA and US Caucasions**

While it was not possible to compare severity of illness directly between OOA and US Caucasians, we were able to assess disease severity indirectly by comparing length of hospital stay and the average number of co-morbidities, which we calculated using the Charlson index, which provides a cumulative co-morbidity score after assigning weights to each diagnosis. [^17^](#_ENREF_17) These analyses revealed that hospital stays were on average shorter among OOA than US Caucasians (4.0 vs. 5.0 days; p < 0.001), and OOA patients had lower Charlson scores (0.95 vs. 1.35; p < 0.001).

**REFERENCES**

**1.** Gallagher TE, Jr., Beiler K (2002) Church Directory of the Lancaster County Amish. Gordonville, PA: Pequea Publishers.

**2.** Tolea M. Patterns of hospital utilization in the Old Order Amish [PhD dissertation] (2007) Baltimore: Epidemiology and Preventive Medicine, University of Maryland, Baltimore

**3.** Agarwala R, Biesecker LG, Hopkins KA, Francomano CA, Schäffer AA (1998) Software for constructing and verifying pedigrees within large genealogies and an application to the Old Order Amish of Lancaster County. Genome Res 8:211-221.

**4.** Agarwala R, Biesecker LG, Tomlin JF, Schaffer AA (1999) Towards a complete North American Anabaptist genealogy: A systematic approach to merging partially overlapping genealogy resources. Am J Med Genet 86:156-161.

**5.** Agarwala R, Schäffer AA, Tomlin JF (2001) Towards a complete North American Anabaptist Genealogy II: Analysis of inbreeding. Hum Biol 73:533-545.

**6.** Agarwala R, Biesecker LG, Schäffer AA (2003) Anabaptist genealogy database. Am J Med Genet 121C:32-37.

**7.** Lee WJ, Pollin TI, O'Connell JR, Agarwala R, Schäffer AA (2010) PedHunter 2.0 and its usage to characterize the founder structure of the Old Order Amish of Lancaster County. BMC Med Genet 11:68.

**8.** Beiler K (1998). Fisher Family History: Descendants and History of Christian Fisher (1757-1838). Vol Third 3rd ed. Ronk's, PA: Eby's Quality Printing

**9.** Gingerich HF, Kreider RW (2002) Amish and Amish Mennonite Genealogies. Vol 2nd Printing. Gordonville, PA: Pequea Publishers

**10.** Beiler K, ed (2009) Descendants and History of Christian Fisher (1757-1838). 4th ed. Grand Rapids, MI: HeuleGordon

**11.** Hsueh WC, Mitchell BD, Aburomia R, et al (2000) Diabetes in the Old Order Amish: Characterization and heritability analysis of the Amish Family Diabetes Study Diab Care 23:595-601.

**12.** Post W, Bielak LF, Ryan KA, et al. (1007) Determinants of coronary artery and aortic calcification in the Old Order Amish. Circulation 115:717-724.

**13.** Streeten EA, Ryan KA, McBride DJ, Pollin TI, Shuldiner AR, Mitchell BD (2005) The relationship between parity and bone mineral density in women characterized by a homogeneous lifestyle and high parity. J Clin Endocrinol Metab 90:4536-4541.

**14.** Mitchell BD, McArdle PF, Shen H, et al. (2008) The genetic response to short-term interventions affecting cardiovascular function: rationale and design of the Heredity and Phenotype Intervention (HAPI) Heart Study. Am Heart J 155:823-828.

**15.** Hurt LS, Ronsmans C, Thomas SL (2006) The effect of number of births on women's mortality: Systematic review of the evidence for women who have completed their childbearing. Population Studies. 60:55-71.

**16.** Green A, Beral V, Moser K (1988) Mortality in women in relation to their childbearing history. BMJ 297:391-395.

**17.** Charlson ME, Pompei P, Ales KL, MacKenzie CR (1987) A new method of classifying prognostic comorbidity in longitudinal studies: development and validation. J Chronic Dis 40:373-383.
